# Correction: The effect of COVID rehabilitation for ongoing symptoms Post HOSPitalisation with COVID-19 (PHOSP-R): protocol for a randomised parallel group controlled trial on behalf of the PHOSP consortium

**DOI:** 10.1186/s13063-023-07132-3

**Published:** 2023-02-07

**Authors:** Enya Daynes, Molly Baldwin, Neil J. Greening, Thomas Yates, Nicolette C. Bishop, George Mills, Matthew Roberts, Malik Hamrouni, Tatiana Plekhanova, Ioannis Vogiatzis, Carlos Echevarria, Rashmita Nathu, Hamish J. C. McAuley, Lorna Latimer, Jennifer Glennie, Francesca Chambers, Ruth Penfold, Emily Hume, Dimitrios Megaritis, Charikleia Alexiou, Sebastian Potthof, Mitchell James Hogg, Catherine Haighton, Bethany Nichol, Olivia C. Leavy, Matthew Richardson, Omer Elneima, Amisha Singapuri, Marco Sereno, Ruth M. Saunders, Victoria C. Harris, Claire M. Nolan, Charlotte Bolton, Linzy Houchen-Wolloff, Ewen M. Harrison, Nazir Lone, Jennifer Quint, James D. Chalmers, Ling-Pei Ho, Alex Horsley, Michael Marks, Krisnah Poinasamy, Betty Ramen, Louise V. Wain, Christopher Brightling, William D.-C. Man, Rachael Evans, Sally J. Singh

**Affiliations:** 1 NIHR Leicester Biomedical Research Centre-Respiratory, Leicester, UK; 2 Department of Respiratory Sciences, University of Leicester, Leicester, UK; 3 NIHR Leicester Biomedical Research Centre- Diabetes, Leicester, UK; 4 Diabetes Research Centre, College of Life Sciences, University of Leicester, Leicester, UK; 5 National Centre for Sport and Exercise Medicine, School of Sport, Exercise and Health Sciences, Loughborough University, Loughborough, UK; 6 Faculty of Health and Life Sciences, Northumbria University Newcastle, Newcastle upon Tyne, UK; 7 The Newcastle upon Tyne Hospitals NHS Foundation Trust, Newcastle, UK; 8 Department of Social Work, Education, and Community Wellbeing, Northumbria University Newcastle, Newcastle upon Tyne, UK; 9 Department of Health Sciences, University of Leicester, Leicester, UK; 10 College of Health, Medicine and Life Sciences, Brunel University, London, UK; 11 Harefield Respiratory Research Group, Guy’s and St Thomas’ NHS Foundation Trust, London, UK; 12 School of Medicine, The University of Nottingham, Nottingham, UK; 13 Centre for Medical Informatics, The Usher Institute, University of Edinburgh, Edinburgh, UK; 14 Usher Institute, University of Edinburgh, Edinburgh, UK; 15 National Heart and Lung Institute, Imperial College London, London, UK; 16 Royal Infirmary of Edinburgh, NHS Lothian, Edinburgh, UK; 17 MRC Human Immunology Unit, University of Oxford, Oxford, UK; 18 Division of Infection, Immunity & Respiratory Medicine, Faculty of Biology, Medicine and Health, University of Manchester, Manchester, UK; 19 Hospital for Tropical Diseases, University College London Hospitals, London, UK; 20 Division of Infection & Immunity, University College London, London, UK; 21 Asthma UK and British Lung Foundation, London, UK; 22 Radcliffe Department of Medicine, University of Oxford, Oxford, UK; 23 University of Dundee, Ninewells Hospital and Medical School, Dundee, UK; 24 Harefield Respiratory Research Group, Heart, Lung and Critical Care Clinical Group, Guy’s and St Thomas’ NHS Foundation Trust, London, UK

**Correction: Trials 24, 61 (2023)**

**https://doi.org/10.1186/s13063-023-07093-7**

Following publication of the original article [1], it was noticed that the author name Dimitrios Megaritis was incorrectly written as Dimitrios Magaritis.

The author group has been updated above and the original article has been corrected.
